# State Laws Supporting Adapted Physical Education Access and Quality for Students With Disabilities

**DOI:** 10.1111/josh.70228

**Published:** 2026-09-17

**Authors:** Elizabeth Piekarz‐Porter, Rebecca M. Schermbeck, Julien Leider, Frank M. Perna, Jamie F. Chriqui

**Affiliations:** ^1^ School of Public Health University of Illinois Chicago Chicago Illinois USA; ^2^ University of Illinois Chicago School of Law Chicago Illinois USA; ^3^ Institute for Health Research and Policy University of Illinois Chicago Chicago Illinois USA; ^4^ Health Behaviors Research Branch (HBRB), Behavioral Research Program (BRP), Division of Cancer Control and Population Sciences (DCCPS) National Cancer Institute Rockville Maryland USA

**Keywords:** adapted physical education, physical activity, physical education, policy surveillance, state law

## Abstract

**Background:**

Children with disabilities are advised to meet the *Physical Activity Guidelines for Americans* and be as active as possible. A key opportunity for physical activity (PA) is physical education (PE).

**Methods:**

State laws (50 states and DC) as of December 31, 2023, were compiled from commercial legal databases and double‐coded as part of the National Cancer Institute's CLASS database. Laws were coded for any adapted physical education (APE) provision, APE teacher certification and qualification, and PE exemptions.

**Results:**

While 49 states addressed the availability of APE, 16 states' laws did not indicate who is eligible. A total of 13 states' laws addressed APE curriculum standards, and 30 addressed APE teacher certification. In total, 15 states addressed who teaches APE at the elementary level and 14 at the middle/high school levels. PE exemptions for disability varied by grade.

**Implications for School Health Policy and Practice:**

State laws represent an opportunity to better address APE, including who is qualified to teach APE and what standards could be used to guide such instruction when it falls to general PE educators.

**Conclusions:**

Greater attention to how state laws address the inclusion of students with disabilities is necessary, as PA is a key component of a healthy lifestyle for all children.

## 1 Introduction

The current *Physical Activity Guidelines for Americans* recommend that children and adolescents ages 6–17 years participate in at least 60 min of moderate‐to‐vigorous physical activity (PA) daily [1]. The Guidelines also state that “children and adolescents with disabilities should meet the key guidelines” and “should be as active as possible and avoid being inactive.” [1] However, according to data from the 2022–2023 National Survey of Children's Health, only 16% of children aged 6–17 years with special health care needs participated in PA for at least 60 min a day [2].

The rationale and benefits behind the *Physical Activity Guidelines* have been well researched and documented. PA contributes to better overall health through improved bone and muscle strength, reduced risk of chronic disease, and reduced social and emotional stress [3, 4, 5]. In addition, research consistently shows that active students are better learners with better academic performance, greater ability to focus, and lower rates of absenteeism [6, 7]. Yet, these PA benefits are not as often promoted to children with disabilities, including those related to chronic disease. For example, childhood cancer survivors are well known to engage in less PA due in part to fatigue and deconditioning that accompanies cancer treatment, as well as new physical mobility limitations and impairments in fitness and muscle functioning that may occur and persist following treatment [8, 9, 10, 11].

A key mechanism for children to receive PA and learn about its benefits is through school physical education (PE) programs. PE itself is a cornerstone of the CDC Comprehensive School Physical Activity Program [6, 12, 13]. PE provides standards‐based curricula and instruction, develops knowledge and fitness for life, and leads to physically active lifestyles in the future [6, 12, 14]. For children with disabilities, clinical care and physical therapy may be offered initially, but over time school and community supports are often the best place to continue physical progress [8, 10].

Under federal law, a free appropriate public education (FAPE) must be available to all children, including children with disabilities [15]. As part of this requirement, each state must ensure that PE, specially designed if necessary, is made available to every child with a disability receiving FAPE [16]. Such adaptations are determined when developing an Individualized Education Program (IEP) for a student, a complicated series of steps that are undertaken by school district personnel and parents working in coordination to determine student goals, milestones, and services provided at school [17]. Moreover, the federal language in this area is broad. For example, the Individuals with Disabilities Education Improvement Act of 2004 (IDEA) requires that a “qualified professional” provide services, without detailing what it means to be “qualified” [18]. As a result, states and school districts are given wide latitude in fulfilling federal requirements. At times, some flexibility may be necessary, for example, during teacher shortages [19, 20]. However, guardrails can also be necessary to ensure that children with disabilities are not shortchanged because of budget constraints [21].

Adapted physical education (APE) is “specially designed instruction in PE that has been adapted or modified so that it is as appropriate for the person with a disability as it is for a person without a disability” [22]. This term will be used throughout this work as it is that which is preferred by the field. Because under the law, APE is considered a specially designed instructional service, it is a required component of a student's education. Moreover, the benefits that children experience because of participation in adapted PA are far‐reaching, including but not limited to expanded aerobic capacity, improved gross motor function and muscle strength, and improved self‐esteem [23, 24]. The Adapted Physical Education National Standards (APENS) were created with the hope of providing students who require APE the full benefit of the instruction to which they are entitled [25]. The APENS are a set of 15 standards of specialized knowledge for APE teachers that build to APENS certification [26]. Developed by the National Consortium for Physical Education for Individuals with Disabilities, these standards also aim to fill the gap left by IDEA regarding who is a “qualified professional” to teach APE. The certification test itself is 100 questions and does require a fee. Within the field, there is some debate as to whether passing a test is sufficient or the extent to which additional professional development should be required for instructors [27].

Under IDEA, students with disabilities must be educated in the “least restrictive environment,” which means with their peers in general education classrooms to the maximum extent possible [28]. As a result, general PE teachers often end up providing APE to students who need it [29]. In those cases, since PE is a required component of special education, the general PE teacher should be included as a member of the IEP team [30], though this is not always the case. When state laws are silent on APE, the result is a patchwork of APE implementation in general PE classrooms. Inclusive and APE often leave students with disabilities experiencing less motor engagement than their peers without disability [31], which may be due in part to variability in state laws governing APE in schools or a lack of guidance for general PE teachers who are providing the instruction. Moreover, PE exemption policies often allow for students to be exempt from PE based on disability status [32]. With the knowledge that PE is an important piece of providing PA opportunities and skills to all children and of the role that states play in interpreting federal requirements in this space [33], it is necessary to understand the details of state APE requirements, standards, and certification. The purpose of this work is to investigate the extent to which teachers are provided with training and opportunities to be prepared to provide APE instruction within general PE based on certification opportunities and requirements that certified teachers are the ones who teach APE.

## 2 Methods

### 2.1 Participants

State statutes and administrative regulations (“state laws”) were collected for all 50 states and the District of Columbia (“states”) in effect as of December 31, 2023. Boolean terms and connector searches were run in two commercial databases—LexisAdvance [34] and WestlawNext [35]. In addition, non‐codified state PE standards were collected when incorporated by reference into state law through searches of each state's Department or Board of Education websites. Search terms included combinations of the words APE, disability, accommodation, modify, inclusive, assist, special education, “Free and Appropriate Public Education” and “Individuals with Disabilities Act.” Laws were collected if they addressed APE and included issues of curriculum standards, teacher certification, or PE exemptions.

### 2.2 Instrumentation

All laws and PE standards were scored by two trained coders (one master's level, the other an attorney) utilizing the ordinal coding scheme (see Table S1) developed as part of the National Cancer Institute's Classification of Laws Associated with School Students (CLASS) database (class.cancer.gov) [36]. Coders reached 99% inter‐coder agreement on the first 10 states coded during the 2023 CLASS data collection period. Still, all laws were double‐coded and compared for consistency. Consensus was reached by the two coders on any identified discrepancy in the remainder of the 41 states.

### 2.3 Data Analysis

Consistent with previous research [37, 38, 39], coding was collapsed as no policy, weak policy, or strong policy (see Table S1 for original and condensed coding schemes). In brief, strong laws are definitively required and meet or exceed federal or national standards, whereas weak laws did not meet the federal or national standards, were weaker, included vague requirements, or were only recommended.

## 3 Results

While 49 states address the availability of APE, the way in which they do so differs significantly (see Table 1). A total of 14 states' laws simply restate the federal definitional requirements related to providing APE, and two states' laws require that APE be made available to every child who needs it (a total of 16 “weak” laws). A total of 33 states' laws include the Federal FAPE language but then also provide for who qualifies for APE, even if just in reference to an IEP (“strong” laws).

**TABLE 1:** State laws regarding adapted PE requirements, 2023 (*N* = 51).

	*n* (%)
Adapted PE requirement	
Not addressed	2 (4%)
Recommends APE for those who may require it, provides a definition of special education which includes APE, or requires that APE be made available without providing a benchmark for qualification (Weak policy)	16 (31%)
Meets Federal FAPE requirements, provides that APE be made available for those who need it, and references IEP or criteria for who might qualify (Strong policy)	33 (65%)

In total, 13 states' laws either include APE curriculum standards or address APE within the general state PE standards (see Table 2). Only three states have strong laws providing that APE certification exists and requires at least a minor in APE (at least 15 credits). A total of 27 states' laws are weak and require less than a minor, only recommend APE certification, or else address elements of APE within general PE certification requirements. And 21 states' laws fail to address certification for APE teachers. Across grade levels, six states' laws have strong policies requiring that all teachers have an APE license to provide APE. Another eight to nine states' laws only require newly hired teachers to have a license or recommend licensing for APE. Still, 36 to 37 states fail to address the qualifications related to teaching APE.

**TABLE 2:** State laws regarding curriculum standards and teacher requirements for adapted PE, 2023 (*N* = 51).

	All grades, *n* (%)	Elementary, *n* (%)	Middle/high, *n* (%)
Addresses APE curriculum standards^a^			
No	38 (75%)		
Yes (standalone or within general PE standards)	13 (25%)		
Certification requirements for APE teachers^b^			
Not addressed	21 (41%)		
Weak policy	27 (53%)		
Strong policy	3 (6%)		
Addresses who teaches APE^c^			
Not addressed		36 (71%)	37 (73%)
Weak policy		9 (18%)	8 (16%)
Strong policy		6 (12%)	6 (12%)

^a^ Laws were not coded separately by grade level.

^b^ There were no differences by grade level, and so results are combined.

^c^ There were no differences by MS/HS and so results are combined.

Finally, 14 states' laws allow for a PE exemption for students based on disability status at the elementary school level, 13 states' laws allow for PE exemption for middle school students, and 21 states' laws provide for a PE exemption for high school students (see Table 3).

**TABLE 3:** State laws allowing exemptions from PE based on disability, 2023 (*N* = 51).

	*n* (%)
Elementary	14 (27%)
Middle	13 (25%)
High	21 (41%)

## 4 Discussion

This study provides important insight into the incongruities in state law attention to APE. By its very nature, APE needs to be adaptable and flexible to the needs of participating students. However, it is still possible to be specific regarding who is entitled to services, who is qualified to teach, and the establishment of basic curriculum standards—provisions that are often not addressed in state laws. General PE laws in 48 states very clearly require that all children participate in PE [36]. Moreover, a previous collection of these laws as part of CLASS shows that most provide for specific time requirements that indicate the number of minutes per week, or days per week, that PE is provided [36, 40]. Prior research shows that schools were more likely to provide PE when they had a strong state law mandating PE time [33]. Alternatively, when states had laws that addressed PE waivers, exemptions, or substitutions for school sports, other school activities, community sports, fitness test scores, or vocational training, schools were more likely to allow such practices as alternatives to PE [41]. Reasonably then, strong state laws that support and protect APE stand to provide a solid framework for schools to follow. Without this backbone, students who would otherwise benefit from APE may not have access to it. And, since most often APE is provided within the general PE setting, clarity in the process, procedures, and training mechanisms becomes vital to support those providing the instruction.

Yet, this analysis shows that the needs of students with disabilities are often not addressed, as 16 states' laws fail to include any information related to who qualifies for APE and how. Often, children receive APE based on decisions made in IEP meetings, making it imperative that PE teachers are included in the IEP process [31, 42, 43]. The development of IEPs is a complicated process that many parents struggle to navigate [44, 45]. Although there are tools and resources available to help guide the conversation [46], often the most successful plans are made with the help of an attorney, which not every family is able to afford [44, 47]. At the same time, IEP plans may direct their focus to traditional academic subjects, even though federal law considers PE to be a direct (not related) service [16, 48] and state laws require PE in 48 of the 51 states [32]. Thus, the existence of strong state laws for APE is important as reference materials in IEPs, and as research in general PE suggests [33], schools may be more likely to adopt comprehensive APE instruction in the presence of strong state law. The development of laws that provide clear references to APE (33 states), either regarding IEP plans or in terms of when APE may be appropriate, can help parents and teachers be aware of the tools with which to help children receive the full benefits of the education to which they are entitled.

Similarly, it is key that there is a mechanism to ensure that state PE exemptions for disability are used only when necessary. In 2014, the CDC found that 85.7% of schools had specific practices related to exemptions for PE based on a long‐term physical or medical disability [49]. However, more research needs to be done to determine how many of these students may actually benefit from APE instead. Schools and teachers should be given the tools to promote the opportunity of PA as the default, navigating any disability as necessary, instead of exempting students from PE.

Notably, nationwide teacher shortages exist in all subject areas [50]. More than ever, schools are lacking qualified teachers for both general and APE [51]. However, only approximately half of all states provide a pathway to APE certification, with under a quarter even addressing whether those who teach APE have any specific training. APENS certification was created with the intention of filling this gap. A study conducted in 2009 investigated caseloads and job demographics of APE educators who were APENS certified and found that factors that potentially influence caseloads include state and school district policies [52]. It then becomes clear that states also need to recognize, promote, and offer certification opportunities with the expectation that general teachers who provide APE be trained to provide APE. Since research shows that students who receive APE within general education settings often end up with less motor skill development than their peers [31], this is a perfect opportunity to lift APE to the platform necessary to ensure that adapting PE leads to meaningful participation and physical movement. As some states have already done, a simple way may be to add the APE requirements into already well‐established general PE certification standards.

It is also notable that while most states provide at least some state‐level guidance on the development, adoption, or creation of general PE standards for school districts to follow, the same attention and guidance is not paid to building out or scaffolding standards for APE. There are two ways that APE can be integrated here. First, states could choose to develop APE standards that can similarly be used as a guide for schools and school districts. For example, Maine has a webpage dedicated to APE, with direct links to its APE endorsement and the APENS [53].

Alternatively, states could update general PE standards to address opportunities for adaptability. CLASS (class.cancer.gov) includes legal citation references, which allows for easy identification of relevant full policy text as described here. In Mississippi, the general PE standards include specific content strands for APE [54]. Oklahoma's law, which incorporates their state‐board general PE standards, specifically requires that “the subject matter standards for physical education: meet the needs of students of all physical ability levels, including students who have a disability, chronic health program, or other special need that precludes the student from participating in regular physical education instruction but who might be able to participate in physical education that is suitably adapted and, if applicable, included in the student's individualized education program” [55]. And in Rhode Island, the general PE standards simply state, “the standards are for all students, regardless of ability. Obvious modifications will be made in lessons, assessments, and procedures for students requiring [Adapted] Physical Education. These students may not progress along the K–12 continuum at the same pace as other students. They can, however, meet the standards when an appropriate [adapted] curriculum that allows for modifications to lessons, assessments, and timing is instituted” [56]. Most states make regular updates to their state PE standards; some simple acknowledgments of making PE adaptable can propel the topic to visibility and provide general education teachers strategies to better integrate APE for students who need accommodations.

### 4.1 Limitations

It is important to note that this descriptive study captures 1 year of codified law. It does not measure implementation of these policies and does not include efforts that may be occurring in local school district policies or within schools individually. Future research may consider investigating the extent to which PA is promoted for children with disabilities through local policies or in combination with community organizations and hospitals.

### 4.2 Implications for School Health Policy and Practice

PE is a key contributor to children's PA, a central component of a healthy lifestyle for all children, including those with disabilities and those recovering from qualifying medical treatments or surgeries, such as for cancer. A FAPE includes access to PE, yet for many states, APE is an afterthought. As general PE teachers are often the ones providing APE, state laws could work to address APE, including who is qualified to teach APE as well as what standards could be used to guide such instruction—particularly when it falls in the general PE setting. In addition, states can support and promote open access to standards, trainings, and qualifying exams, such as APENS, to help teachers become qualified in providing the instruction that they have been tasked with under federal law.

## 5 Conclusions

There is an opportunity for states to elevate APE within law in ways that will provide greater support for students and teachers alike. A PE classroom that teaches the abilities of all students and provides access for PA together will help build lifelong healthy habits for students with and without disabilities.

## Funding

This work was supported by Westat Inc. (Subcontract no. 6632.01‐S04) and the National Cancer Institute.

## Ethics Statement

The authors have nothing to report.

## Conflicts of Interest

The authors declare no conflicts of interest.

## Supporting information


**Table S1:** Coding tool for scoring relevant state laws, 2023.

## Data Availability

The data that support the findings of this study are openly available on the CLASS website at class.cancer.gov.
