# Supplementary material for: State Laws Supporting Adapted Physical Education Access and Quality for Students With Disabilities
Source: J Sch Health. 2026 Sep 17;96(10):e70228. doi: 10.1111/josh.70228 (PMC13584841; doi:10.1111/josh.70228)
Supplement: Supplementary file 1 [file JOSH-96-0-s001.docx]

Supplementary Table 1. Coding Tool for scoring relevant state laws, 2023

| **Policy Area** | **Description** | **Score** | **Collapsed Coding** | **Description of Score** |
| --- | --- | --- | --- | --- |
| Adapted PE requirements | Score reflects the extent to which state laws meet the federal requirements to make physical education available to every child receiving a free appropriate public education | 0 | No law | No law |
|  |  | 1 | Weak law | Recommends adapted PE for those who may require it or provides a definition of special education which includes PE |
|  |  | 2 |  | Requires that adapted PE be made available to every child who needs it, without providing a benchmark for qualification |
|  |  | 3 | Strong law | Meets Federal FAPE requirements and provides that adapted PE must be made available to every child who needs it, including referencing IEP or other criteria/definition of who might qualify for adapted or specially designed PE |
| PE curriculum standards | Enhancing factors that address the extent to which state law references adapted PE standards | 0 | NA | No law |
|  |  | 1 |  | Applies if state addresses adapted PE within general PE standards or has adopted stand-alone adapted PE standards/guidelines |
| Adapted PE certification | Reflects the degree to which state law addresses the educational degree and certification requirements for adapted PE teachers | 0 | No law | No law |
|  |  | 1 | Weak law | Recommends certification / licensure / endorsement or includes adapted PE content as part of the general PE certification requirements |
|  |  | 2 |  | Offers certification / licensure / endorsement to teach adapted PE and requires preparation that is less rigorous than a college minor (e.g. less than 15 credit hours) in adapted PE |
|  |  | 3 | Strong law | Offers certification / licensure / endorsement to teach adapted PE and requires a college minor (or a minimum of 15 credit hours) in adapted PE |
|  |  | 4 |  | Offers certification / licensure /endorsement to teach adapted PE and requires a college major (or a minimum of 30 credit hours) in adapted PE |
| Adapted PE teacher requirement | Reflects the degree to which state law requires adapted PE to be taught by a licensed/credentialed/endorsed adapted physical education teacher | 0 | No law | No law |
|  |  | 1 | Weak law | Recommends that teachers be licensed/credentialed/endorsed in adapted PE to teach adapted PE |
|  |  | 2 |  | Requires that newly hired teachers be licensed/credentialed/endorsed in adapted PE to teach adapted PE |
|  |  | 3 | Strong law | Requires that all teachers be licensed/credentialed/endorsed in adapted PE to teach adapted PE |
